# 5′ UTR Control of Native ERG and of Tmprss2:ERG Variants Activity in Prostate Cancer

**DOI:** 10.1371/journal.pone.0049721

**Published:** 2013-03-05

**Authors:** Francesca Zammarchi, George Boutsalis, Luca Cartegni

**Affiliations:** 1 Department of Molecular Pharmacology and Chemistry, Memorial Sloan-Kettering Cancer Center, New York, New York, United States of America; 2 Department of Pharmacology, Weill Cornell Medical College of Cornell University, New York, New York, United States of America; 3 Experimental Therapeutic Center, Memorial Sloan-Kettering Cancer Center, New York, New York, United States of America; UMDNJ-New Jersey Medical School, United States of America

## Abstract

ERG, a member of the ETS transcription factor family, is frequently overexpressed in prostate cancer as a result of its fusion to the androgen-responsive Tmprss2 gene. Different genomic rearrangements and alternative splicing events around the junction region lead to multiple combination of Tmprss2:ERG fusion transcripts that correlate with different tumor aggressiveness, but their specific functions and biological activities are still unclear. The complexity of ERG expression pattern is compounded by the use of alternative promoters, splice sites, polyadenylation sites and translation initiation sites in both the native and fusion contexts. Our systematic characterization of native ERG and Tmprss2:ERG variants reveals that their different oncogenic potential is impacted by the status of the Ets domain and the configuration of the 5′ UTR region. In particular, expression and activity of functional ERG and Tmprss2:ERG variants are influenced both by translation initiation signals within the different isoforms and by inhibitory upstream Open Reading Frames (uORF) in their 5′ UTRs. Stable expression of ERG and Tmprss2:ERG variants promoted cell migration/invasion, induced a block of proliferation and induced a senescence-like state, suggesting a role for these variants in the prostate tumorigenesis process. In addition to Tmprss2:ERG fusion products, a group of related native ERG isoforms is also highly over-expressed in fusion-carrying prostate cancers, and share the same translation initiation site (in ERG exon 4) with the commonly observed Tmprss2 exon1 joined to ERG exon 4 (T1:E4) fusion-derived variant. Usage of this ATG can be preferentially down-regulated by directed antisense-based compounds, possibly representing the basis of a targeted approach that distinguishes between tumor–associated and normal ERG.

## Introduction

Chromosomal translocations are well established key events in the development of hematological malignancies and sarcomas [1]. The identification of recurrent translocations between the androgen-responsive Tmprss2 gene and members of the Ets family of transcription factors in prostate cancer (PCa) has changed the panorama of PCa biology [2].

More than 80% of PCa samples harbor oncogenic fusions, most commonly involving the 5′ region of the Tmprss2 locus (with its promoter) joined to Open Reading Frame (ORF) of various Ets genes [3], although other combinations have been described [4], [5], [6]. The single most common rearrangement, Tmprss2 exon1 joined to ERG exon 4 (T1:E4, or variant III) is present in ∼50% of PCa cases [3].

Ets gene fusions play a role in the transition from prostatic intraepithelial neoplasia (PIN) to adenocarcinoma and are associated to aggressive lesions and poor prognosis [7], [8], [9], [10], [11]. Overexpression of ERG in prostate cell lines activates cell invasion programs and results in the development of PINs in mice, but cannot drive carcinogenesis [5], [8], [10], [12], as cooperation with separate genetic lesions, for example pTEN loss, is needed to trigger progression to advanced disease [7], [13], [14].

Several variants of the normal ERG gene product have been described, arising from a combination of alternative splicing, polyadenylation and transcriptional initiation [15], [16], [17], [18]. The specifics of the genomic rearrangements also introduce considerable structural heterogeneity in the 5′ region. In addition to the common T1:E4 transcript, Tmprss2 exons 1, 2 and 3 can be combined with ERG exons 2, 3, 4, 5 and 6 in various alternative splicing patterns that can generate at least 17 distinct Tmprss2:ERG transcripts [2], [19], [20], [21]. These can serve as markers for disease progression and correlate to the aggressiveness of the tumors [8], [9], [19]. In particular, the T2:E4 variant (Tmprss2-exon2:ERG-exon4 or variant VI), where the native ATG in Tmprss2 exon 2 is in frame with the ERG ORF, is associated with pathological and clinical aspects of aggressive disease [19].

The mechanistic basis for the different oncogenic potential of the fusion isoforms remains to be elucidated, and it could be related to intrinsic differences in the N-terminal regions or to the effect(s) that variation in the 5′ region of the mRNA can have on RNA stability or expression.

To test this hypothesis, we assessed the use of 3 alternative promoters, 2 common alternative splicing events and 3 polyadenylation sites (PAS) in normal tissues relative to Tmprss2:ERG-expressing prostate tumors. These independently regulated events combine to generate 30 ‘main’ native isoforms, some of which we found to be also highly overexpressed in tumors. The characterization of the translation initiation sites used by the most common native ERG and fusion variants reveals that the specific organization of the 5′ UTR region is one of the principal determinants of their biological activity and identifies an ATG in exon 4 as a putative target for antisense-based translation inhibition in prostate cancer.

## Results and Discussion

### Structure of the ERG Gene

Multiple native ERG isoforms can arise due to a combination of alternative promoters, splicing and polyadenylation. These isoforms can in turn combine with Tmprss2 and other 5′ partners to produce numerous ERG-derived variants, with variable prognostic values [8], [9], [19]. To better understand the activities of ERG-derived oncogenic products, we sought to initially clarify the ERG gene structure and to characterize its expression pattern.

There are considerable inconsistencies in the published ERG nomenclature, with at least four different classification schemes, with the obvious consequence of generating confusion in understanding and comparing data from different studies (e.g.: the large terminal exon that contains the Ets and transactivation domains is variably referred to as exon 11 [2], [22], 12 [3], 16 [18], [23], 17 [24]).

**Figure 1:**
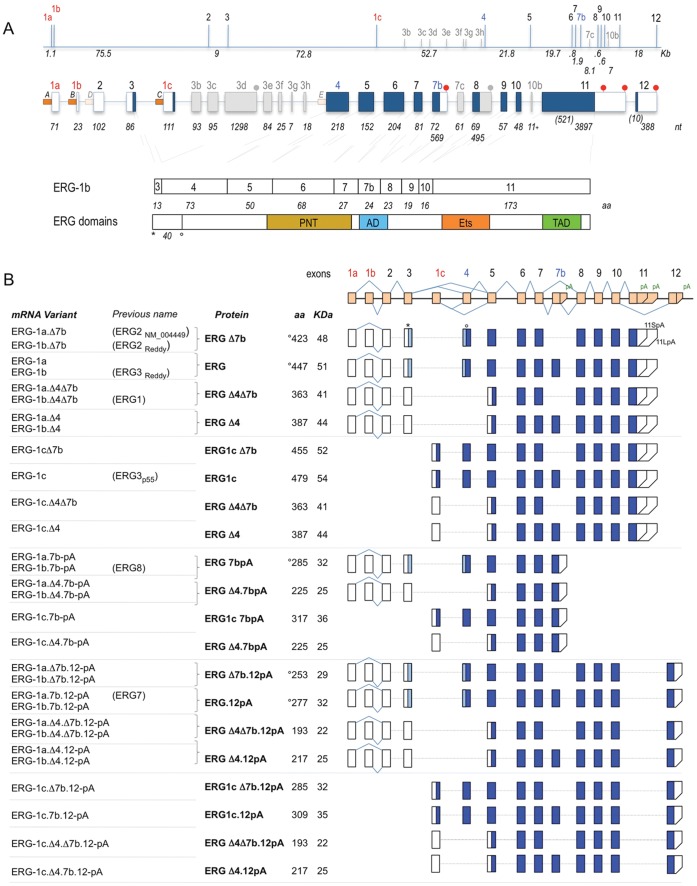
Human ERG gene structure and main isoforms. (**A**) Top: The ∼300 Kb human ERG locus, drawn roughly to scale. Approximate intron sizes are indicated, along with exons position (bars). Red indicates first exons, blue common alternative ones and gray uncommon ones. Middle: Exon structure, with exon sizes at the bottom. Blue boxes indicate the main predicted ORFs, white boxes the untranslated regions and gray the uncommon exons. Red circles indicate polyA sites. Bottom: alignment of the exons forming the main ORF (ERG-1b) with the protein’s domains. Numbers indicate size in amino acids. PNT = pointed domain, AD = alternative domain, Ets = Ets domain, TAD = transactivation domain. Asterisk and circle indicate position of the first and second ATG. (**B**) Human ERG main variants. Alignment of exons forming the 30 main RNA variants of human ERG. Blue indicates the ORF, light blue the additional region from the ATG in exon 3. For each variant, the proposed name is indicated next to previous nomenclature (if available). The proposed protein name is reported along the predicted size in aa and KDa. Variants derived from the alternative usage of promoter 1a and 1b are paired as they lead to related mRNAs and identical proteins.

We report in Figure 1A an up-to-date view of the exon-intron structure of the ∼300 Kb ERG locus (ENSG000000157554) and propose a unified, rational nomenclature of RNA and protein variants that aims at incorporating the most established conventions. The structure is mainly based on the 11 exons of Refseq entry NM_004449.4 (Uniprot entry P11308 for ERG2), and incorporates the exon numbering used in the seminal Tomlins paper [2], [22] first describing the Tmprss2:ERG fusion. In short, we maintained as “exon 4” the 218nt exon that is the main partner of Tmprss2 and as “exon 11” the large 3897nt exon encoding the Ets domain; we named Exon 1a, 1b, 1c the three mutually exclusive ‘first’ exons following the three validated promoters P_A_, P_B_ and P_C_. To ensure the rational inclusion of additional exons that might be identified in the future, we distinguished by letters the alternative exons not part of the 11 reference ones. For example, the 72 nt alternative exon included between exons 7 and 8 is called ‘exon 7b’.

We then identified the main alternative regulatory events that generate the majority of ERG variability: 3 alternative promoters (P_A_, P_B_ and P_C_); two common alternative splicing events (inclusion/skipping of exon 4 or 7b); three separate polyA signals (PAS 7bpA, 11LpA and 12pA). Combinatorial usage of the alternative events generates 30 possible ‘major’ ERG transcript variants (Figure 1B), which can encode 15 different predicted ERG-related polypeptides, with 3 different N-terminals, 3 different C-terminals and 1 possible internal variation (inclusion or skipping of 24 aa in the Alternative Domain encoded by Exon 7b).

In addition to the 30 ‘major’ variants described in Figure 1, a plethora of ‘minor’ isoforms have been reported in the literature or are present in databases. The list includes variants showing skipping of exons 2, 5, 7 and 8; usage of a proximal (Short) polyA site in exon 11 (11SpA) or of an additional intronic one downstream of exon 8 (8pA); and inclusion of supplementary alternative exons 7c, 10b and of multiple alternative exons derived from intron 3 (exons 3b–h), indicated in gray in figure 1A. Isoforms ERG4, ERG6 and ERG9 from the previous Owczarek study [18] are minor variants and have been reclassified in this group.

Since any of these additional minor events could combine independently with all the structurally compatible major isoforms, hundreds of variants could potentially be generated. However, these events appear to occur sporadically, and their physiological relevance is unknown.

### ERG Expression: a Cancer-associated Switch in Promoter Usage

**Figure 2:**
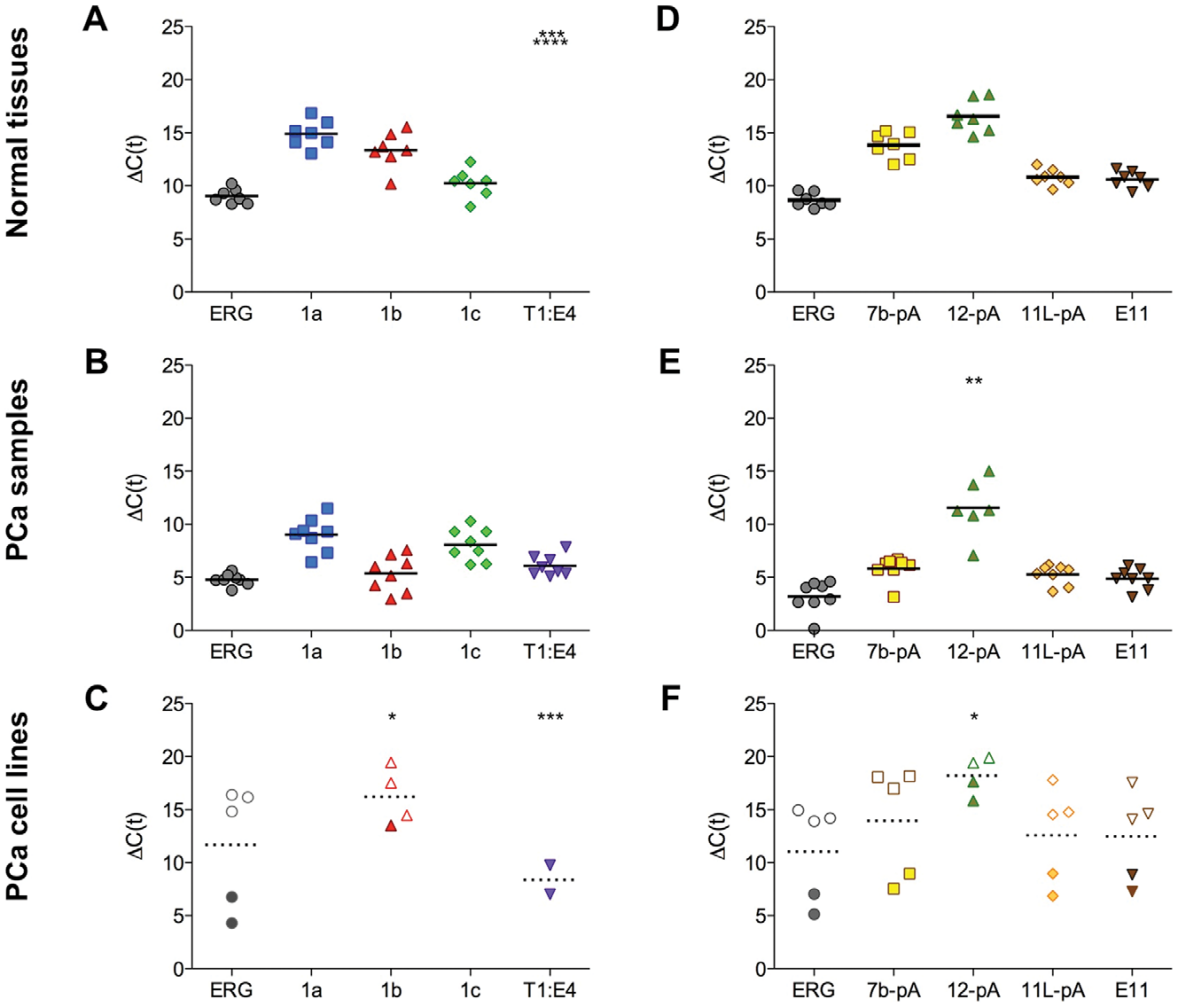
Differential ERG variant expression in normal tissues and PCa cells. Quantification by qPCR of alternative ERG variants in normal tissue (**A, D**), primary PCa samples (**B, E**) and PCa cell lines. (**C, F**). (**A–C**) Quantification of promoter usage. Primer sets specific for the 3 different ERG promoters and for the Tmprss2:ERG fusion where used, along with a primer set spanning exons 5–7 to quantify total ERG. Normal tissues do not express the fusion product. Expression of variants 1a and 1c is virtually undetectable in the PCa cell lines analyzed. For panel C, open symbols indicate LnCap, DU145 and C4-2 (not expressing Tmprss2:ERG fusion), full symbols indicate VCap and NCI-H660 (expressing Tmprss2:ERG fusion). See also Fig. S1 A–C. (**D–F**) Quantification by qPCR of alternative polyadenylation usage. Primer sets specific for the 3 different polyA sites where used, along with a primer set to quantify total ERG and one set to quantify total exon 11 levels in order to infer 11SpA usage. See also Fig. S1 D–F. In all cases, each indicated value represents averages of ≥3 independent experiments and is presented as ΔC(t) normalized to the housekeeping gene GAPDH, therefore a “high” ΔC(t) value means low levels of expression and a “low” value means high level of expression. Horizontal bars indicate the mean. Asterisks indicate that the product was not detected in the sample and would therefore be equivalent to an experimental point at the top of the table, but it is not reflected by the mean.

We set out to investigate usage of promoters, PAS or splicing signals by qPCR analysis of ERG expression in different normal tissues, tumors and cell lines (Figure 2). While some degree of tissue-to-tissue variability is observed (Fig. S1A), in general promoter P_C_ (mean Δc(t) = 10.2) appears to be the most active in normal tissue (including in prostate), ∼25-fold and ∼10-fold more active on average than promoters P_A_ (Δc(t) = 14.9) and P_B_ (Δc(t) = 13.4), respectively (Figure 2A). On the other hand, in a panel of 8 primary PCa samples expressing Tmprss2-ERG fusions, promoter P_B_ (mean Δc(t) = 5.4) accounts for the majority of native ERG transcript and is present at levels comparable to those of the fusion itself (Δc(t) = 6), over 100-fold more abundant than the same transcript in normal prostate (Δc(t) = 12.8) (Figure 2B and Fig. S1A). Indeed, in several Tmprss2:ERG-carrying samples the native P_B_ promoter, rather than the fusion gene, is the principal source of ERG transcript (Fig. S1B). Compared to normal prostate, promoters P_A_ (Δc(t) = 9) and P_C_ (Δc(t) = 8) are also activated, but not to the degree of P_B_. In PCa cell lines, P_B_ is the only active native ERG promoter, with undetectable signals from P_A_ or P_C_ (Figure 2C). Two of the tested PCa cell lines, VCap and NCI-H660, express the Tmprss2:ERG fusion (Figure 2C, full symbols), which is instead absent in PCa cell lines not containing the Tmprss2:ERG fusion (LNCap, DU145, C4-2, open symbols and Fig. S1C). As expected, no expression from any of the native promoters was observed in the NCI-H660 cell line, which carries the fusion on both alleles and lost all endogenous promoters [25].

From this first set of experiments we conclude that a promoter switch occurs in PCa tumors and cell lines and that increased usage of native promoter P_B_ is associate with (and possibly contributes to) the tumor phenotype.

Overexpression of ERG in PCa was previously described [23], but following the discovery of the Tmprss2:ERG fusion, it has been typically ascribed to this event. However, our finding that in addition to the fusion-derived transcripts, some native ERG variants can also be highly overexpressed in PCa, suggests a bigger role for native ERG in PCa development. This is supported by the observation that endogenous mouse *Erg* transcripts are overexpressed in tumors from prostate conditional *Pten* ^−/−^;*Trp53* ^−/−^ mice, compared to *Pten* ^−/−^;*Trp53* ^+/+^ mice [7]. Importantly, while the latter model results in an indolent form of PCa, the former produces an aggressive phenotype [7].

The differential activation of the three native promoters in the fusion-carrying PCa samples suggests that this aberrant expression is transcriptionally regulated. An intriguing possibility is that activation of the native P_B_ promoter may be driven directly or indirectly by ERG itself as part of a positive regulatory loop. For example, several putative c-Myc responsive elements were identified immediately upstream of the ERG P_B_ promoter [18]. Since c-Myc is a key downstream target of ERG [26], the androgen-dependent activation of ERG from the fusion, or a separate PTEN-dependent Myc activation [27] could trigger a self–sustaining ERG/Myc oncogenic loop, which could eventually become androgen-independent. Indeed, consistently with this model, a feed-forward mechanism where expression of endogenous ERG is controlled by overexpression of the fusion product has recently been described [28].

### ERG Expression: Alternative Polyadenylation and Splicing

Analogous to what we did to study the promoter’s usage, we then explored the role of alternative polyadenylation and splicing in the expression of the ERG variants. Of the three principal PAS described (Figure 1), the 11L-pA is needed for a fully functional ERG protein, whereas the PAS in intron 7b (7b-pA) and exon 12 (12-pA) generate C-terminally truncated ERG isoforms lacking the Ets DNA-binding domain and the transactivation domain (TAD).

The 11L-pA PAS was the most commonly used in normal tissues (mean, ΔC(t) = 10.8), about 8-fold more than 7b-pA (ΔC(t) = 13.8) and 50-fold more than 12-pA (ΔC(t) = 16.6) (Fig. 2D). However, in Tmprss2:ERG-expressing PCa samples (ΔC(t) = 5.2), usage of 7b-pA is strongly activated, and becomes about as common as the 11L-pA (ΔC(t) = 5.8) (Figure 2E). The same is true in PCa cell lines expressing the fusion (Figure 2F) suggesting that switching to this pattern of expression could correlate with tumor progression. It is important to note that, while this variant lacks the Ets and TAD domains, it retains the dimerization determinants, and thus its expression might influence the activity of other full-length variants present. A proximal PAS within exon 11 (11S-pA) [15], [18] was indirectly assessed by comparing qPCR of regions on either side of it (E11 and 11L-pA), and similar levels of expression were observed, suggesting that usage of 11S-pA is marginal under most normal and pathological conditions (Figure 2D–F and Figure S1D–F).

Since alternative splicing variants can influence ERG or Tmprss2:ERG fusion functions [24], we analyzed regions around exon 7b and exon 4 in normal tissues, PCa samples and cell lines by semi-quantitative PCR. Both alternative splicing events were readily detectable, with a clear prevalence of the exon 4 and exon 7b inclusion variants. However, we didn’t observe any significant enrichment in the relative amount of either splicing variants in PCa samples, suggesting that the modulation of these two specific splicing events may not play a determinant role in PCa.

Altogether, these data indicate that the full-length products, containing exon 7b and the Ets and TAD domains, are the most abundant ERG variants regardless of the status of the upstream regions, both in normal tissues and tumors. The strong increase of the truncated TE:7bpA isoform relative to the full-length variant could simply reflect changes in the splicing/polyadenylation machinery of cancer cells, but it could also indicate its (yet unexplored) role in ERG biology in tumors.

### N-terminal Heterogeneity of ERG Isoforms

**Figure 3:**
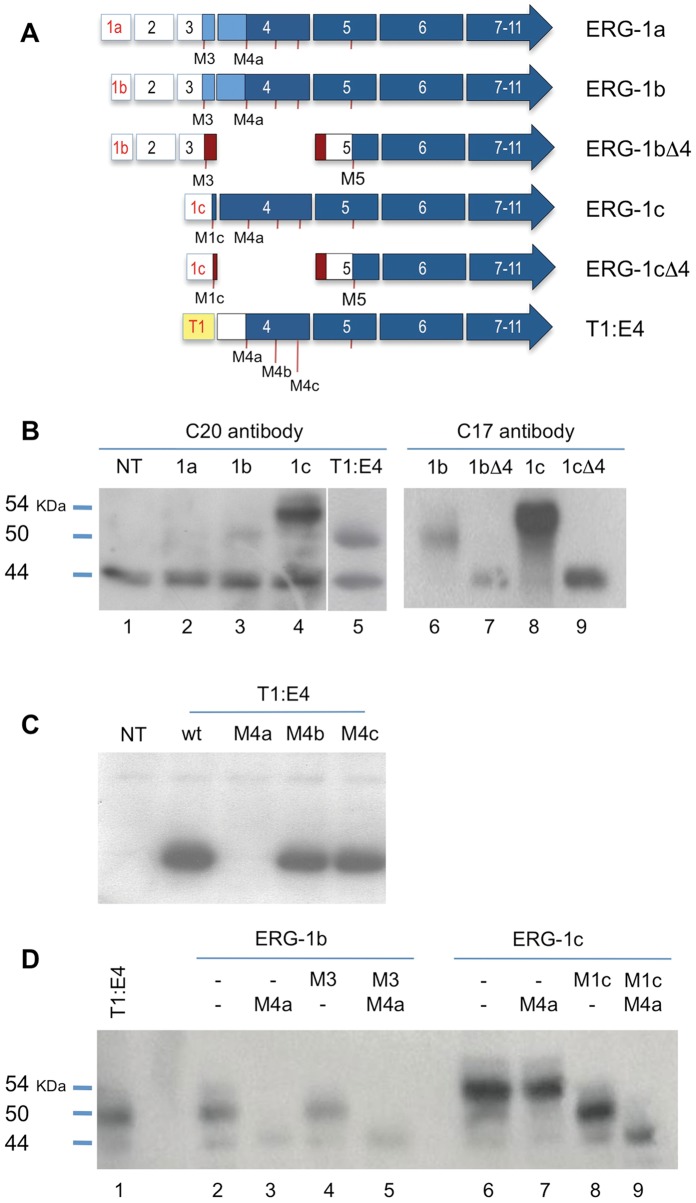
Mapping of translation initiation of ERG isoforms. (**A**) N-terminal heterogeneity in human native ERG variants. The 5′ regions for the indicated variants are reproduced. Boxes represent exons with the ORF in blue. Red ticks below the exons indicate in-frame ATGs in exons 1c and exons 3 to 5. The out-of-frame products caused by exon skipping, from the otherwise in frame ATGs are indicated in red. (**B**) Western Blot (WB) of transient expression of the variants in HeLa cells. Antibody C20 recognizes exogenous protein and an endogenous band at ∼44 KDa corresponding in size to Δ4 variants. Antibody C17 preferentially recognizes exogenous ERG bands. (**C**) Mutations that independently eliminate the 3 in-frame ATGs in exon 4 were introduced into the cDNA of T1:E4 and analyzed as above. (**D**), Similarly, mutations were introduced to eliminate the ATGs in exon 3 (ERG-1b) or in exon 1c (ERG-1c) by themselves or in combination with mutations in the first in-frame ATG (M4a) in exon 4 (ERG-1b and ERG-1c).

To evaluate whether the described heterogeneity in the 5′ region affect ERG expression and activity, we sub-cloned the native variants ERG-1a, ERG-1b, ERG-1c, ERG-1b.Δ4, ERG-1c.Δ4, and the common fusion variant T1:E4, with their complete 5′ UTRs and with exon 7b included (Fig. 3A). Upon transient transfection in HeLa cells, ERG-1c cDNA efficiently expressed the expected 54 KDa product (Fig. 3B, lane 4). On the contrary, expression of the native ERG-1a and ERG-1b variants was inefficient, resulting in peptides smaller than the ∼55 KDa expected if the first in-frame ATG in exon 3 was used (Fig. 3B, lanes 2–3). Interestingly, this peptide co-migrated with that generated by the transient over-expression of the T1:E4 fusion variant, at ∼50 KDa (lane 5). Additional variation in the N-terminal region derives from exon 4 skipping, which alters ERG’s main ORF so that the predicted starting ATGs in exon 1c or exon 3 would not be able to generate an ERG-related peptide (Figure 3A). Transient overexpression of ERG-1b.Δ4 or ERG-1c.Δ4 both resulted in ERG-related peptides migrating at ∼44 KDa, consistent with the usage of an in-frame M5 ATG in exon 5 (Figure 3B, lanes 7,9). This is most evident when using ERG antibody C-17, which readily recognizes recombinant ERG, but not the endogenous proteins. A different ERG antibody (C-20) preferentially recognizes an endogenous band corresponding in size to ERGΔ4 (lanes 1–5), making the switch to M5 usage harder to detect.

Since T1:E4 lacks the predicted initiation codons from both Tmprss2 and ERG transcripts, an alternative internal ATG from within ERG’s open reading frame must be used to express an ERG-related product from the fusion mRNA, likely from exon 4. To identify the T1:E4 initiation codon, a series of methionine-to-alanine point mutations were generated for the three in-frame ATG codons present in ERG exon 4 (Fig. 3A). Mutation at nucleotides 79 (M4a), but not 121 (M4b) and 184 (M4c) of exon 4 abolished T1:E4 expression (Figure 3C), indicating that the fusion transcript uses the first in-frame ATG for expression of the ERG peptide.

To map the starting ATGs from the native ERG isoforms similar mutations where also introduced alone or in combination in the first in-frame ATGs in exon 3, 1c and 4 (Figure 3D). Mutation of the ATG in exon 3 (M3) didn’t have any effect on the expression of the ∼50 KDa product (Figure 3d, lanes 2 vs. 4), while mutation of the next ATG in exon 4 (M4a) abrogated it both in the wt and M3 context (Figure 3D, lanes 3 and 5), indicating that ERG-1b (and ERG-1a) do not use their first in-frame ATG, as would be predicted by a 5′cap-dependent scanning model of translation initiation [29]. Instead, usage of the following ATG in exon 4 generates a peptide identical to that encoded by the T1:E4 fusion. On the contrary, when the start codon in exon 1c is mutated (M1c), ERG-1c mobility is reduced from ∼54 KDa to ∼50 KDa (Figure 3D, lanes 6 vs. 8), like T1:E4 and ERG-1b (lane 1 and 2), consistent with a switch to the M4a ATG. Indeed, mutation of this ATG results in the abrogation of the ∼50 KDa product in favor of a still smaller product.

### Biological Characterization of the ERG Isoforms

**Figure 4:**
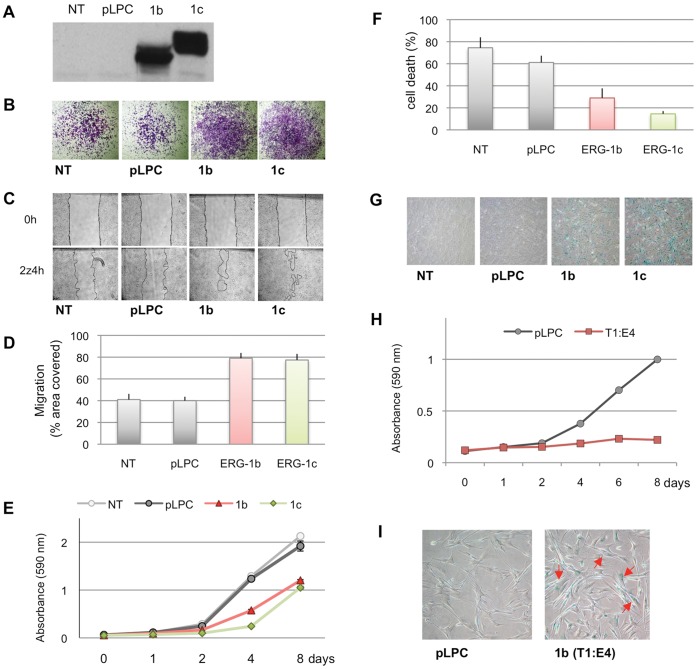
Biological activity of ERG-1b and ERG-1c. (**A**) NIH-3T3 cells stably expressing high–level of ERG-1b and ERG-1c were analyzed as in Fig 3. (**B–C**) Following drug selection, ERG-overexpressing clones (1b and 1c), empty-vector control (pLPC) and untreated NIH-3T3 cells (NT) were assayed for their invasion potential using a matrigel invasion assay (B), and in a standard wound scratch assay to measure their motility (C). (**D**) Quantification of (C), using ImageJ software. Bars indicate standard deviation. (**E**) Growth curve of NIH-3T3 stable clones. Following selection, cells were fixed at 48 h intervals over a period of 8 days and stained with crystal violet. (**F**) After drug selection, NIH-3T3 stable clones were plated and serum starved, and cell death was measured by trypan blue method. (**G**) 5 days after drug selection NIH-3T3 stable clones were stained for SA-β-galactosidase. (**H**) Growth curve of IMR90 cells stably expressing high-level of ERG1b/T1:E4 or the empty vector. After drug selection, cells were fixed at 48 h intervals over a period of 8 days and stained with crystal violet. (**I**) 5 days after drug selection IMR90 stable clones were stained for SA-β-galactosidase. Red arrows indicate bi-nucleated cells. Representative images are shown in B, C, G and I.

To assess whether the structural N-terminal differences between the cancer-associated ERG-1b/T1:E4 and the normal ERG-1c affect intrinsically their biological activity, we initially stably expressed their corresponding cDNAs in NIH-3T3 cells (Figure 4A) and selected clones with robust expression. In agreement with previous reports, we observed promotion of cell invasion and migration by both ERG variants, as assayed by trans-well migration through matrigel (Figure 4B) and scratch wound assay (Figure 4C–D). The extent of the effect on migration/invasion was comparable in ERG-1b and ERG-1c indicating that they are similarly active, at least in some basic aspects of their biology.

Surprisingly, expression of ERG variants in this context resulted in decreased cellular proliferation (Figure 4E). This was not associated with cell death. On the contrary, expression of ERG-1b and especially ERG-1c resulted in protection from cell death following serum starvation (Figure 4F). This behavior could reflect the activation of an oncogene-dependent senescence-like state by ERG expression as previously described for other oncogenes [30]. Indeed, ERG-expressing cells, but not controls, stained positive for senescence biomarker (beta)-Galactosidase (Figure 4G). Intrigued by these results we subsequently stably overexpressed the ERG-1b/T1:E4 isoform and the corresponding empty vector in the normal human fibroblast cell line IMR90, a well-characterized cell model system for cellular senescence. As with the NIH-3T3 cells, over-expression of the ERG-1b/T1:E4 isoform resulted in increased cell migration and cell invasion, (Figure S2). Moreover, IMR90 cells over-expressing the ERG-1b/T1:E4 isoform showed senescence-like phenotypes such as a inhibition of cellular proliferation (Figure 4H), accumulation of bi-nucleated cells and elevated SA-β-gal activity, a classical biomarker of senescence (Figure 4I).

This result is particularly exciting in light of the fact that the senescence program is activated once a cell has sensed a critical level of damage or dysfunction, pointing to a critical role for ERG-1b/T1:E4 overexpression as an initial event leading to PCa. Moreover it is important to note that cellular senescence can be detected in early-stage human PCa specimens and can be triggered by acute loss of PTEN in a p53-dependent fashion in mouse PCa models [31], suggesting that ERG activation might result in an initial senescent phenotype which requires subsequent environmental or genetic changes to progress to malignancy.

### ATG Context and uORFs Affect ERG Translation Efficiency and Functions

**Figure 5:**
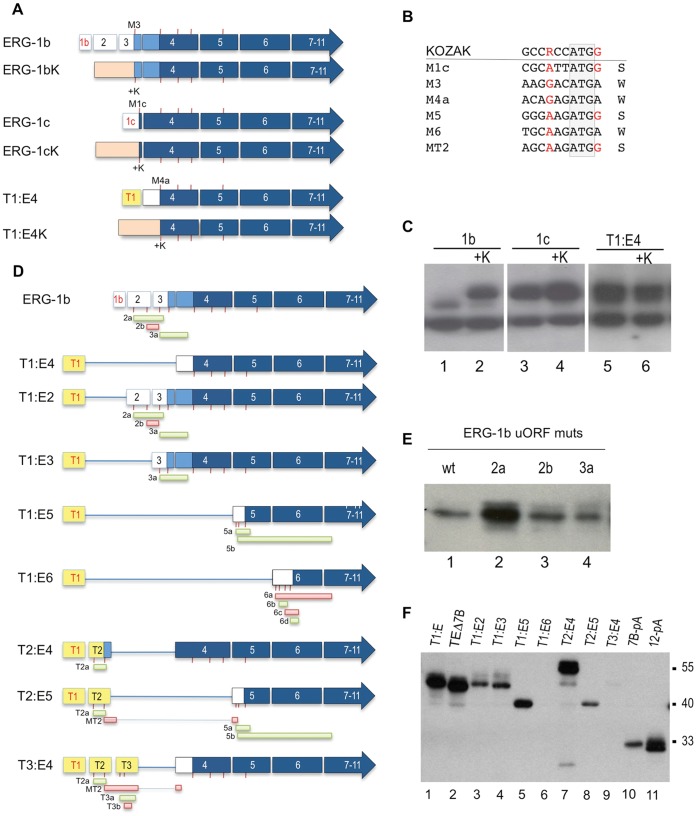
Role of the 5′ UTR region in ERG and Tmprss2:ERG variants expression. (**A**) The native 5′ UTR of ERG-1b, ERG-1c and T1:E4 were replaced with a common one from the expression vector (in orange), and an optimized Kozak sequence. The replaced ATGs are in exon 3 (M3), 1c (M1c) and 4 (M4a) (**B**) Context of multiple in-frame ATG used as start codons in various ERG and Tmprss2:ERG variants. The sequence around the ATG is aligned to a consensus Kozak sequence, with the important conserved positions at -3 (R) and +4 (G) highlighted in red (counting the A as +1). Context is evaluated as ‘strong’ (S) if both positions are conserved, and ‘weak’ (W) if they are not. An expanded analysis of ATGs in the 5′UTR region of ERG and Tmprss2:ERG is reported in Table S1. (**C**) WB analysis of the variants and mutants represented in (A). Improving the M3 context favors its use at the expenses of the M4a ATG. (**D**) uORFs that might influence translation efficiency of ERG variants in the 5′ UTRs of ERG-1b and several common Tmprss2:ERG variants. Red ticks indicate ATGs, boxes below indicate the putative uORF generated and their approximate length (drawing not to scale). Red boxes indicate strong ATG contexts and green weak ones (as defined in (B)). More details about the uORFs are listed in Table S1. (**E**) Effect of independently mutating uORFs’ ATG in ERG-1b: mutation of the first ATG in exon 2 releases suppression of translation and increases levels of ERG from the ATG in exon 4. (**F**) Expression of ERG and Tmprss2:ERG variants**.** Full-length cDNAs for the variants, including their entire 5′ UTRs, were expressed and lysates from transiently transfected HeLa cells were analyzed by western blot.

Efficiently translated eukaryotic mRNAs require an optimal context around the start codon, to aid in its recognition by the ribosome (the Kozak sequence: GCCRCC**ATG** G) [32]. However, this is not always sufficient to explain translation efficiency. For example, although ERG-1a/b and T1:E4 share the same ATG in exon 4 (M4a) to initiate translation, their expression levels are different (Figure 3B lanes 2,3 and 5) suggesting a role for their different 5′ UTRs. Indeed, substitution of the natural 5′ UTRs with one containing a consensus Kozak (Figure 5A-B) led to activation of expression from the M3 ATG in the ERG-1b variant, with synthesis of the originally predicted 55 KDa peptide (Figure 5C, lanes 1–2). This confirms that elements within the 5′ UTR of ERG-1b inhibit its translation and it rules out that the low ERG-1b (M3) abundance is due to an intrinsic instability of its N-terminal domain.

Upstream Open Reading Frames (uORFs) are typically short ORFs that start within the 5′ UTR, are out-of frame with the main downstream coding sequence and can reduce its expression [33], [34]. While no uORFs are present in ERG-1c or in T1:E4, three uORFs exist in the ERG-1b 5′UTR (Figure 5D, top), which could interfere with recognition of the predicted ATG of ERG-1b (Table S1). Abrogation of the uORF starting at uM2a by mutation of the ATG to GCG led to increased ERG-1b expression (Figure 5E, lane 2), indicating that the engagement of the scanning ribosome by the first encountered ATG, to generate a short 29 aa uM2a peptide, is a limiting factor in ERG-1b expression from the downstream ATG.

The presence of uORFs could also similarly affect the levels of expression of the various androgen-driven Tmprss2:ERG fusion proteins, with prognostic implication, since the 5′ heterogeneity in fusion is associated with different clinical outcomes [19], [20]. The different pathological profiles could be due to changes in biological activity depending on the primary protein sequence or to differences in its abundance, *via* modulation of translation. Such variations might explain how, for example, the T2:E4 fusion is associated with a more aggressive phenotype [19], [20].

To investigate whether translation efficiency plays a role in the activity of the fusion variants and to characterize their biological properties, we subcloned the 8 most common TMPRSS:ERG variants (T1:E2, T1:E3, T1:E4, T1:E5, T1:E6, T2:E4, T2:E5, T3:E4) [19], [20], along with the exon 7b skipping variant in the common T1:E4 context (TEΔ7b), and the two C-terminal truncated variants (TE7bpA and TE12pA). All variants were transiently expressed in HeLa cells under identical conditions (Figure 5F).

Expression of the 54 KDa peptide from the T2:E4 isoform, which is associated with an aggressive phenotype, is robust (Figure 5F, lane7), as expected from a transcript that contains a strong native translation initiation site (MT2, Figure 5B). The presence of a weak overlapping uORF (uMT2a) is not sufficient to reduce T2:E4 expression. Isoforms T1:E2 and T1:E3 include the native ERG ATG on exon 3 (M3), and in principle should be efficiently expressed. However, as for the native ERG variants ERG-1a and ERG-1b, the M3 ATG is not used. Instead, usage of the M4 ATG in exon 4 leads to generation of the same ∼50 KDa product described for ERG-1b. Similarly to ERG-1b, T1:E2 low expression levels might be due to the presence of the 3 uORF depicted in Figure 5D. Indeed T1:E3, which lacks exon 2 and the uORFs it carries, has a slight but reproducibly higher level of expression than T1:E2 (Figure 5F, lanes 4 vs. 3). Isoform T1:E5 uses the good Kozak in exon 5 and is expressed efficiently, despite the presence of two uORFs (5a and 5b). The same 44 KDa protein is also generated by T2:E5, but less efficiently, due to the presence of two additional uORFs (T2a and MT2). A similar scenario plays out for the T3:E4 variant tested, which uses the weak ATG in exon 4. In this case, the presence of 4 inhibitory uORFs almost completely abrogates translation of the ∼50 KDa product. Skipping of exon 5 generates the rare isoform T1:E6, a putative protein lacking the PNT domain but containing the Ets and transactivation domain. However, its expression is suppressed due to the weakness of the Kozak associated to the in-frame ATG in exon 6, and the presence of 4 inhibitory uORFs.

From these studies we can conclude that overall, the levels of transient expression of Tmprss2:ERG isoforms appear to correlate with their 5′ UTR context. The strength of the Kozak sequence and the number and relative strength of the uORFs both contribute to translation efficiency of the ERG and Tmprss2:ERG variants, and should be taken into consideration as an additional layer of complexity when assessing the oncogenic potential of the fusion variants observed in clinical samples.

**Figure 6:**
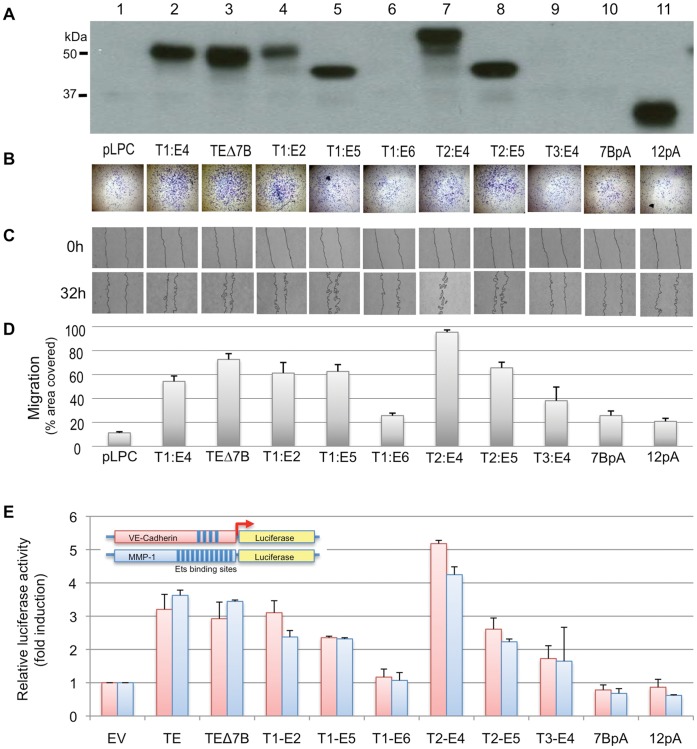
Activity of ERG and Tmprss2:ERG variants. (**A**) Stable expression of ERG variants in NIH-3T3 cells. Expression was analyzed as in Fig 3. (**B–C**) Cells from (A) were assayed for their invasion potential using a matrigel invasion assay (B) and for their motility using a wound healing assay (C). Representative images are shown. (**D**) Quantification of (C), using ImageJ software (≥4 independent experiments). Bars indicate standard deviations. (**E**) Transient transcriptional activation of ERG-dependent promoters. Firefly luciferase cDNA was cloned downstream of the VE-Cadherin or MMP-1 promoters as depicted. The VE-Cadherin (red) and the MMP-1 (blue) promoters contain respectively 4 and 11 Ets binding sites (blue boxes, not to scale). Luciferase reporters were transiently co-expressed in HeLa cells with Tmprss2:ERG variants. A renilla luciferase construct was used to normalize for variation in transfection efficiency. Dual-luciferase assay was performed and activity is represented as fold-activity over that of co-transfected empty vector. Data represent averages of at least 3 independent experiments, with bars indicating standard deviations.

Indeed, in invasion and wound healing assays in NIH-3T3 cells stably expressing multiple fusion isoforms, we noticed that cell mobility correlated to the expression levels of the various isoforms (Figure 6A–D). The same type of correlation was apparent in transient co-transfection experiments (Figure 6E), where we measured the ability of the different isoforms to trans-activate a luciferase reporter under the control of either the VE-cadherin or the matrix metalloproteinase-1 (MMP-1) promoters, which are well established ERG targets [35], [36].

Two additional isoforms, TE:7bpA and TE:12pA, which lack Ets and TAD functional domains, where included in the above analysis because they could potentially display modifying or even dominant-negative properties on ERG activity. Additionally, TE:7bpA RNA is overrepresented in tumors. However, TE:7bpA is expressed at very low levels both in transient and stable experiments (Figure 5F, lane 10 and Figure 6A, lane 10), possibly because of intrinsic instability, making it impossible to reach any conclusion on its biological role. On the other hand, the robust expression of the structurally similar TE:12pA does not affect the rate of migration (Figure 6). In addition, it does not drive expression of luciferase from the VE-Cadherin or the MMP1 promoters, nor it can interfere with the activity of full-length variants in promoting such expression (Figure S3). Thus, at least under these conditions, this isoform behaves like a null mutant rather than a dominant-negative, and the Ets and/or TAD domains encoded by exon 11 are essential for the activity of ERG variants.

### Specific Inhibition of Oncogenic ERG Variants

**Figure 7:**
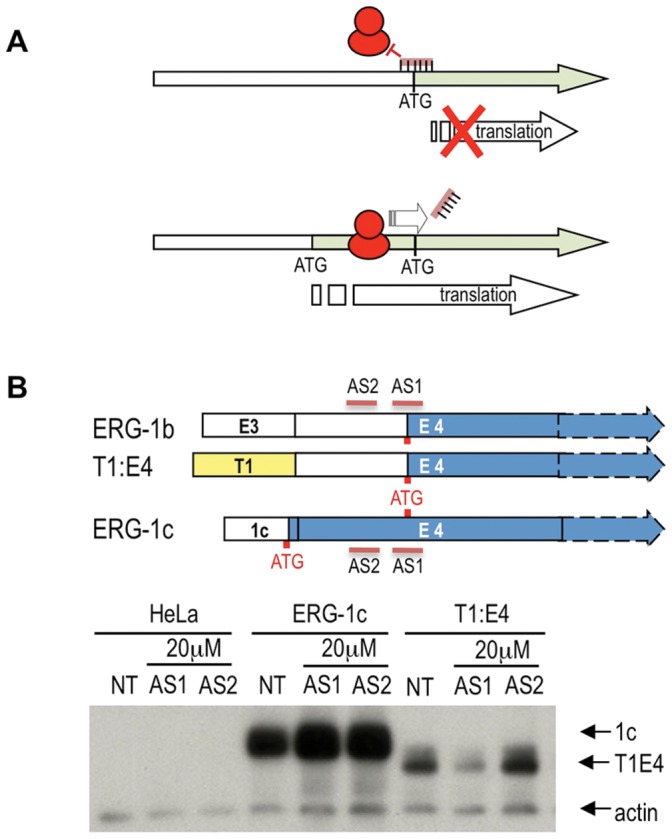
Specific inhibition of oncogenic ERG by blocking the ERG exon 4 ATG. (**A**) Morpholinos base-paired to the translation initiation site can prevent its efficient recognition by the ribosome. However, the translating ribosome displaces the bound antisense oligomers if it can start translating from a separate ATG upstream. (**B**) A morpholino compound (AS1) is targeted to the ATG in exon 4 used by the T1:E4 and ERG-1b cancer-associated variants. A control morpholino compound (AS2) is targeted to sequences upstream, in the exon 4 UTR region. Only the AS1 compound inhibits transient T1:E4 expression in HeLa cells. On the other hand, neither AS1 nor AS2 have any effect on translation starting from the upstream ATG on exon 1c when ERG-1c is transfected, even though the binding site for the compounds are the same as in T1:E4.

We have shown that both the common PCa fusion variant T1:E4 and the native PCa-overexpressed variant ERG-1b use the same ATG in exon 4, whereas the variant most abundant in normal tissues uses one in exon 1c. This raises the possibility to specifically target the cancer-associated ATG, without interference with the normally expressed ERG isoforms. To this end, we decide to use phosphorodiamidate morpholino oligomers (morpholinos), a class of antisense compounds that can be used *in vitro* and *in vivo* to modulate gene expression by interfering with splicing patterns, miRNA maturation or translation [37], [38]. Morpholinos targeted directly at the translation initiation site can inhibit translation by hindering recognition by the ribosome [37]. On the other hand, compounds targeted downstream of the ATG are easily displaced by the translating ribosome and are thus ineffective (Fig. 7A). In the case of ERG, a morpholino targeted to the exon 4 ATG should thus prevent its usage but not that of an upstream ATG, such as for example that on exon 1c. Indeed, a compound (AS1) designed to bind to the region containing the ATG on exon 4 reduced expression of the T1:E4 variant in transient transfection experiments, whereas a similar compound (AS2) targeted to a region upstream did not (Figure 7B). Most importantly, neither had any effect on the transient expression of ERG-1c, the most commonly expressed variant in non-tumor tissues (Figure 7B).

Although further testing of these compounds are beyond the scope of the present study, they suggest that development of specific translation-blocking compounds that effectively and selectively reduce the levels of aberrantly expressed ERG isoforms without affecting normally expressed ones is feasible. Such compounds will serve as an important toolset to further our understanding of a pathway that is improperly activated in the majority of prostate cancers, and could form the basis of a future therapeutic approach.

In summary, the work presented here helps elucidating the complex scenario of ERG and tmprss2:ERG fusion variants by rationally organizing and characterize ERG variants. Furthermore, the observation that a specific subset of native (non-fusion) ERG transcripts is strongly activated in tumors, shifts the focus back to what contribution may come from the endogenous gene and underscores the possibility of a positive-feedback loop involving ERG and Tmprss2-ERG, which could conceivably lead to androgen-independence.

The identification of controlling 5′UTR elements, in particular the translation initiation context and the presence and characteristics of uORFs, furthers our comprehension of the correlation between expressed variants and pathological characteristics. Besides, a correct understanding of what polypeptides are actually expressed from the different mRNAs would inform both in vitro and in vivo experiments with model animal systems.

Finally, we present proof-of-principle evidence that the cancer-associated oncogenic ERG variants could be pharmacologically inhibited without disturbing the functions of the ‘normal’ variants expressed in non-tumor tissues. An approach that could be in principle applied to other such oncogenic fusions.

## Materials and Methods

### Ethics Statement

All patients provided written informed consent and samples were procured and the study was conducted under Memorial Sloan-Kettering Cancer Center Institutional Review Board approval.

### Samples

Total RNAs from prostate tissues expressing Tmprss2:ERG fusion transcripts were a generous gift from the late Dr. W. Gerald and were collected with the written consent from all participants, in accordance with MSKCC approved IRB protocols. Total RNAs from the normal panel of tissues were from Clontech (Human RNA Master Panel II).

### Tissue Culture

All cell lines used were obtained from ATCC (Manassas, VA). HeLa and VCap were grown in DMEM+10% FBS; LnCap in RPMI-1640+10% FBS; PC3 in F-12K+10% FBS, 1.5 g/L sodium bicarbonate; DU145 in EMEM-NEAA +10% FBS, 1 nM sodium pyruvate, 1.5 g/L sodium bicarbonate; NCI-H660 in HITES media with 5% FBS.

### PCR

Total RNA was extracted from cancer cell lines using Trizol (Invitrogen). Contaminating genomic DNA was removed by TURBO DNA-free (Ambion). cDNAs were generated using SuperScript III First-Strand Synthesis System (Invitrogen). RT-PCRs were performed for 35 cycles (30s@95°C, 30s@60°C, 90s@72°C), using cDNA derived from 45 ng of retrotranscribed RNA (RNA-equivalents), 0.2 µM primers and Platinum *Taq* Pol (Invitrogen). qPCR were performed using ∼24 ng RNA-equivalents from the normal tissues or PCa cell lines, or ∼2.4 ng RNA-equivalents from the PCA samples and Platinum SYBR Green qPCR Supermix (Invitrogen). The mean cycle threshold (C_t_) for each sample was normalized to the expression of GAPDH. Primers from IDT.

### Primer Sequences

Tmprss2-ex.1-F GGTACCATGACCGCGTCCTCC

ERG-ex.1a-F CCCCCGAGGGACATGAGA

ERG-ex.1b-F CCGACGGCGGCGCTAACC

ERG-ex.1c-F GCTCTAAACAACCTCATCAAAACT

ERG-ex.1b-Kozak-F CCGCTCGAGCTCGCCACCATGATTCAGACTGTCCCG

ERG-ex.1c-Kozak-F CCGCTCGAGCTCGCCACCATGGCCAGCACTATTAAGG

ERG-ex.6-F GACGACTTCCAGAGGCTCAC

ERG-ex.10-F AAGTAGCCGCCTTGCAAAT

ERG-ex.11-F CAGGAGTCTGCATTTGCACT

ERG-ex.3-R AGCCCATCTACCAGCTGTTCA

ERG-ex.4-R GTAGGCACACTCAAACAACGACTGG

ERG-ex.5-F GCCAAAGGCGGGAAGGCGGTGGGCAGCCCAGAC

ERG-ex.7-R CTGAAGTCAAATGTGGAAGAGG

ERG-ex.7BpA-R GACTGTCTGGGACTGGCTTCAGC

ERG-ex.8-R ATCTCCTGGGGGGCTCATATGGTA

ERG-ex.5-R GTCTGGGCTGCCCACCGCCTTCCCGCCTTTGGC

ERG-ex.11-R GGGGGTCTAGATTATTAGTAGTAAGTGCCCAG

ERG-ex.11-R GCTCTAGAGGGTGCCAAACATCCTATTT

ERG-ex.11L-R AAGAAGGACCTGGAGAGGCT

ERG-ex12pA-R GACCCAGTCCCAGAAGTCAC

GAPDH-F TGCACCACCAACTGCTTAGC

GAPDH-R GGCATGGACTGTGGTCATGAG

1bmut-F GGGGTACCCCGACGGCGGCGCTAACC

1cmut-F GGGGTACCGCTCTAAACAACCTCATCAAAACT

ERG-M2a-F AAAGCAAGACAAGCGACTCACAGAGAAAA

ERG-M2a-R TTTTCTCTGTGAGTCGCTTGTCTTGCTTT

ERG-M2b-F CAGAGAAAAAAGGCGGCAGAACCAAGGGCA

ERG-M2b-R TGCCCTTGGTTCTGCCGCCTTTTTTCTCTG

ERG-M3-F AACAGCTGGTAGGCGGGCTGGCTTACT

ERG-M3-R AGTAAGCCAGCCCGCCTACCAGCTGTT

ERG-M4a-F GGCTAAGACAGAGGCGACCGCGTCCT

ERG-M4a-R AGGACGCGGTCGCCTCTGTCTTAGCC

ERG-M4b-F GACTTCCAAGGCGAGCCCACGCG

ERG-M4b-R CGCGTGGGCTCGCCTTGGAAGTC

ERG-M4c-F AGGGTCACCATCAAAGCGGAATGTAACCCTAGCC

ERG-M4c-R GGCTAGGGTTACATTCCGCTTTGATGGTGACCCT

VE-Cadherin-F GAAGATCTACACTCCCCATCTTGTGCTC

VE-Cadherin-R CCCAAGCTTGAGCGTGAGTGGAGCTCTGT

MMP-1-F GAAGATCTCCTCCTGAAATTCTGGGATTATAG

MMP-1-R CCCAAGCTTACTGGCCTTTGTCTTCTTTCTCAG

### Plasmids and Site-directed Mutagenesis/Exon Deletion Constructs

cDNAs of ERG-1a, -1b, -1bΔ4, -1c, -1cΔ4, T1:E4 and TEΔ7B were amplified from the VCap cell line and subcloned into pcDNA3.1(+) or pLPC retroviral vector. cDNAs include the entire 5′UTR region and the ORF until the stop codon in exon 11 of ERG. cDNAs of the fusion isoforms were all generated by RT-PCR from the prostate tumors (except T1:E5 and T1:E6, generated by exon deletion of T1:E4). Overlap extension strategy was employed for exon deletions and point mutations.

### Transfection Methods of ERG and TE Isoforms

24 hours transient transfections of HeLa cells (5×10^4^ cells/well in 12-well plates) were performed with Fugene 6 (Roche) with 500 ng of DNA: 400 ng plasmid and 100 ng pd2-EGFP (Clontech) to monitor transfection efficiency. pcDNA3.1(+) or pLPC expression vectors alone were used as controls.

### Western Blot Analysis

Hot lysis buffer (1% SDS, 1 mM Na_3_VO_4_, 10 mM Tris, pH 7.4, 95°C) was used to prepare lysates. Protein concentration was determined using the *RCDC Protein Assay Kit II* (Bio-Rad). Denatured samples were run on 10% SDS-PAGE and transferred to PVDF (Millipore). Membranes blocked for 1 h with 5% milk in PBS+0.05% tween20 (PBS-T), then probed overnight at 4°C with primary antibodies: a-ERG C-17 (sc-354, 1∶200, Santa Cruz), a-actin (A4700, 1∶100000, Sigma), a-tubulin (T5158, 1∶100000, Sigma). Membranes were incubated with HRP-conjugated anti-rabbit (NA934V, 1∶1000, GE Healthcare) or anti-mouse (NA931V, 1∶1000, GE Healthcare) secondary antibodies for 1 h. Protein bands visualized with Supersignal West Femto (Thermo Scientific), per manufacturer instructions. Unless otherwise specified, the a-ERG C-17 antibody was used for the detection of ERG and Tmprss2-ERG variants.

### Invasion Assay

Cell culture transwell inserts (24-well format, 8 µM pores, Falcon) were coated with 2 µg Matrigel Basement Membrane Matrix (BD Biosciences). NIH-3T3 and IMR90 stable clones were plated at 1×10^5^ cells/well in serum-free media. Medium containing 10% serum was added to the lower chamber as chemoattractant, and cells were incubated for 6 hs at 37°C, 5%CO_2_. Invading cells on the lower surface of the transwell were fixed with 20% methanol and stained with 0.5% crystal violet.

### Wound Healing Assay

5×10^5^ NIH-3T3 or IMR90 cells were seeded in 6-well plates and starved 16 h in media containing 2% serum. The cell layer was disrupted by introducing a scratch (wound), rinsed with PBS and then fresh media (2%FBS) was added. Cell migration was monitored every 6/8 hours. Quantification of the area covered by migrating cells was assessed with ImageJ software.

### SA-β-galactosidase Staining

5 days after drug selection, transduced cells were fixed in 0.5% glutaraldehyde/PBS for 15 min at room temperature. After fixation, cells were washed twice with PBS and incubated in fresh senescence-associated β-Gal (SA-β-gal) staining solution [5-bromo-4chloro-3-indolyl β-D-galactoside (X-Gal) 1 mg/mL, K3Fe[CN]6 0.21 mg/mL, K4Fe[CN]6 0.16 mg/mL, MgCl2 2 mM] at 37°C without CO_2_ for 6 h (IMR90) or 12 h (NIH-3T3). After SA-β-Gal staining, cells were washed 3X with H_2_O and then cells were photographed.

### Growth Curve

NIH-3T3 and IMR90 cells were infected with the indicated retroviruses. After drug selection, cells were seeded in 48-well plates. Cells were fixed and stained with 1% crystal violet for 30 minutes at room temperature. The plates were then washed extensively and rigorously to remove excess dye and dried. The dye taken up by cells was eluted in 0.1 N HCl for 60 min at 37°C and the A_590_ of the acid-extracted stain was measured on a plate reader (Molecular Devices). Each point of the growth curve experiments was calculated from 4–6 wells.

### Cell Death Experiment

NIH-3T3 and IMR90 cells were infected with the indicated retroviruses. After drug selection, cells were allowed to recover for 24h and seeded on 12-well plates. After 24h, cells were washed with PBS and grown for 24 h in medium containing 0.1% (v/v) FBS. Cells were then trypsinized and dead cells were stained with trypan blue.

### Luciferase Reporter Gene Assay

The VE-cadherin and MMP-1 promoters were PCR amplified from human genomic DNA, and cloned into the pGL3 Luciferase reporter vector (Promega). HeLa cells were seeded at 1.5×10^4^ cells/well in 48-well plates and transfected with 0.5 µl Fugene6, 100ng of the fusion isoform plasmid, 390 ng Firefly reporter construct and 10 ng of Renilla-TK as a co-reporter. 24 h post-transfection, luciferase activity was assessed with the Dual-Luciferase Reporter Assay System (Promega) in a LMaxII reader (Molecular Devices). 50 µl of LARII reagent were added, followed 10 sec later by 30 sec reading time. Stop&Glo (50 µl) reagent was then added to quench the firefly signal for 5 seconds, followed by a 2 sec reading time of the renilla activity.

### Morpholino Treatment

Morpholinos (Gene Tools) were dissolved at 1 mM in 100 mM Tris-HCl (pH 8). HeLa cells were transfected for 3 hs (1×10^6^ cells, 100 mm plate, 6 µg plasmid DNA), trypsinized, seeded in a 24-well plate at 3×10^4^ cells/well and left to adhere for ∼2 hours. 20 µM morpholinos (in complete DMEM) were added first, followed by 6 µM Endoporter (Gene Tools) to facilitate uptake, and incubated for 24 hours. AS1 5′-CGGTCATCTCTGTCTTAGCCAG-3′; AS2 5′-TCCGTAGGCACACTCAAACAAC-3′.

## Supporting Information

Figure S1 **This figure represents an alternative depiction of the data in** **Figure 2** **, to emphasize comparison of data points within samples.** (A–C) Quantification by qPCR of promoter usage. Primer sets specific for the 3 different ERG promoters and for the Tmprss2:ERG fusion where used, along with a primer set spanning exons 5–7 to quantify total ERG. Normal tissues do not express the fusion product. Expression of variants 1a and 1c is virtually undetectable in the PCa cell lines analyzed. For panel C, open symbols indicate LnCap, DU145 and C4-2 (not expressing Tmprss2:ERG fusion), full symbols indicate VCap and NCI-H660 (expressing Tmprss2:ERG fusion). Promoter P_C_ is the most active in normal tissues, while promoter P_B_ is the most active in cancer tissue and the only one active in prostate cancer cell lines. NCI-H660 cells only express ERG from the Tmprss2:ERG fusion because the fusion is present on both alleles and therefore the natural ERG promoters are completely absent. **(D–F)** Quantification by qPCR of alternative polyadenylation usage. Primer sets specific for the 3 different polyA sites where used, along with a primer set to quantify total ERG and one set to quantify total exon 11 levels in order to infer 11SpA usage. The distal PolyA site on exon 11 (11LpA) is the most active in normal tissues, while PolyA site 7b is strongly activated in tumors and in prostate cancer cell lines that carry the fusion. The proximal site on exon 11 (11SpA) is barely used under any circumstance, although the evidence is indirect. **(G)** Approximate location of the primers used for the amplifications. In all cases, each indicated values represent averages of ≥3 independent experiments and is presented as ΔC(t) normalized to the housekeeping gene GAPDH, therefore a “high” ΔC(t) value means low levels of expression and a “low” value means high level of expression. Horizontal bars indicate the mean. Asterisks indicate that the product was not detected in the sample and would therefore be equivalent to an experimental point at the top of the table, but it is not reflected by the mean.
(TIF)

Figure S2 **Effect of ERG 1b/T1:E4 ERG fusion variant on migration and invasion of IMR90 cells.** Following drug selection, ERG 1b/T1:E4 -overexpressing clones or empty-vector control (pLPC) were assayed for their migration and invasion potential using a transwell migration (A) or matrigel invasion assay (B), as detailed in Material and Methods.
(TIF)

Figure S3 **Activity of truncated fusion isoforms lacking Ets and TAD domains.** Transient transcriptional activation of ERG-dependent VE-Cadherin promoter. The luciferase reporter was transiently co-expressed in HeLa cells with full-length T1:E4 or/and truncated TE:7bpA and TE:12pA variants (plus a Renilla luciferase vector to normalize for variation in transfection efficiency). Dual-luciferase assay was performed and activity is represented as fold-activity over that of co-transfected empty vector. Averages of at least 3 independent experiments, with standard deviations, are represented. The truncated isoforms alone cannot induce expression of luciferase, and when co-expressed together with the full-length protein they fail to inhibit its activity, ruling out for them a dominant-negative role. Further experiments would be required to reach more definitive conclusions.
(TIF)

Table S1 **Context and characteristics of uORF and in-frame ATG in ERG variants.** ATGs from the 5′ region of various ERG variants are listed. ATG in frame with ERG are indicated in red and preceded by a ‘M’ ( = Met), ATG resulting in uORF are indicated in black, with the number indicating the exon that harbors them. The predicted length of the ORF/uORF (in amino acids and KDa) is reported, along with the distance (in nucleotides) from the translated ATG, whether the uORF overlaps with the translated ORF, and the ATG context. The context is considered ‘strong’, if both the determinant positions at −3 (G/A) and +4 (G) are conserved, and ‘weak’ if not.
(TIF)
