# Supplementary figures and images for: 5′ UTR Control of Native ERG and of Tmprss2:ERG Variants Activity in Prostate Cancer
Source: PLoS One. 2013 Mar 5;8(3):e49721. doi: 10.1371/journal.pone.0049721 (PMC3589450; doi:10.1371/journal.pone.0049721)

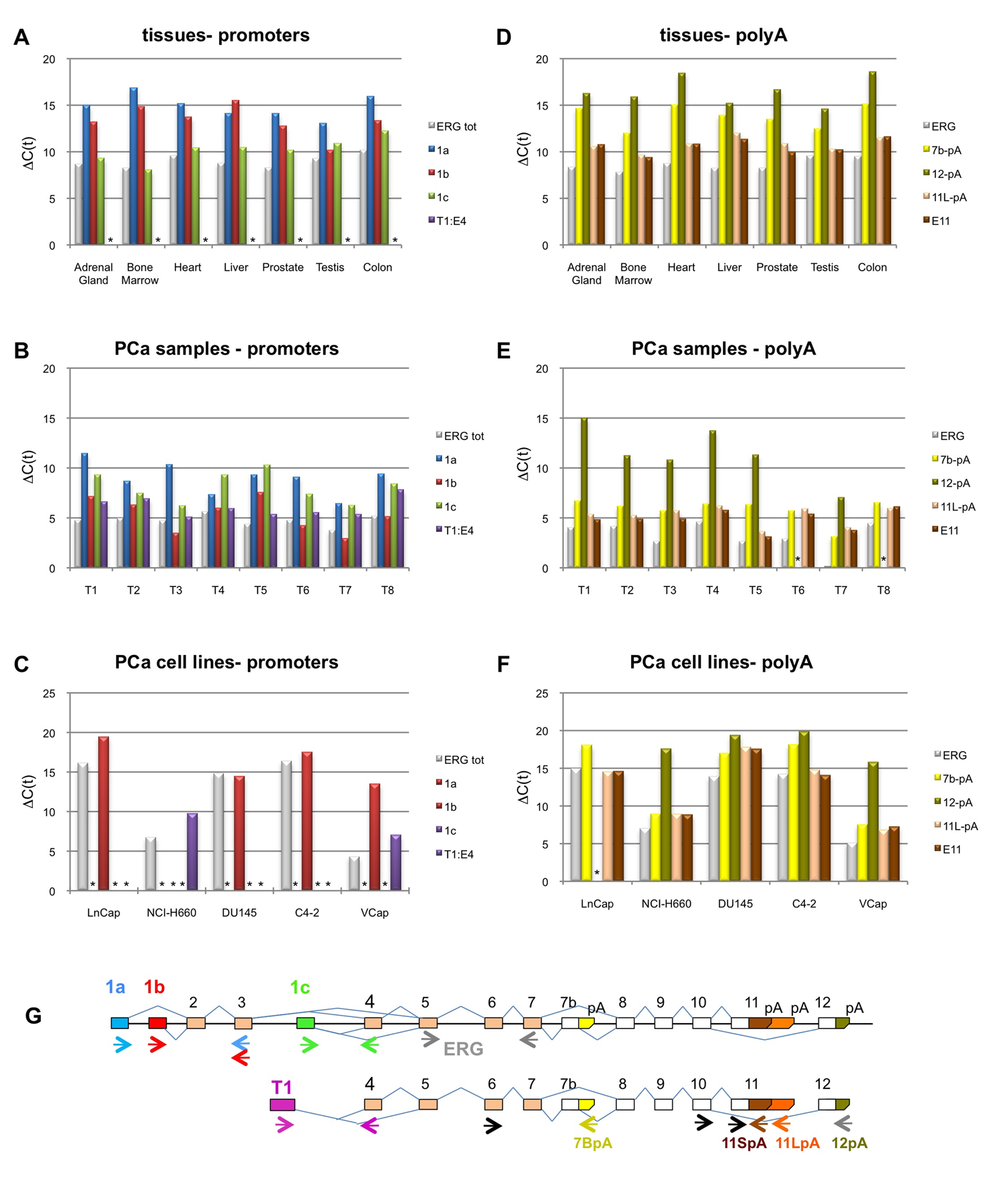

Supplement: Figure S1 — This figure represents an alternative depiction of the data in Figure 2 , to emphasize comparison of data points within samples. (A–C) Quantification by qPCR of promoter usage. Primer sets specific for the 3 different ERG promoters and for the Tmprss2:ERG fusion where used, along with a primer set spanning exons 5–7 to quantify total ERG. Normal tissues do not express the fusion product. Expression of variants 1a and 1c is virtually undetectable in the PCa cell lines analyzed. For panel C, open symbols indicate LnCap, DU145 and C4-2 (not expressing Tmprss2:ERG fusion), full symbols indicate VCap and NCI-H660 (expressing Tmprss2:ERG fusion). Promoter PC is the most active in normal tissues, while promoter PB is the most active in cancer tissue and the only one active in prostate cancer cell lines. NCI-H660 cells only express ERG from the Tmprss2:ERG fusion because the fusion is present on both alleles and therefore the natural ERG promoters are completely absent. (D–F) Quantification by qPCR of alternative polyadenylation usage. Primer sets specific for the 3 different polyA sites where used, along with a primer set to quantify total ERG and one set to quantify total exon 11 levels in order to infer 11SpA usage. The distal PolyA site on exon 11 (11LpA) is the most active in normal tissues, while PolyA site 7b is strongly activated in tumors and in prostate cancer cell lines that carry the fusion. The proximal site on exon 11 (11SpA) is barely used under any circumstance, although the evidence is indirect. (G) Approximate location of the primers used for the amplifications. In all cases, each indicated values represent averages of ≥3 independent experiments and is presented as ΔC(t) normalized to the housekeeping gene GAPDH, therefore a “high” ΔC(t) value means low levels of expression and a “low” value means high level of expression. Horizontal bars indicate the mean. Asterisks indicate that the product was not detected in the sample and would therefore be equivalent [file pone.0049721.s001.tif]

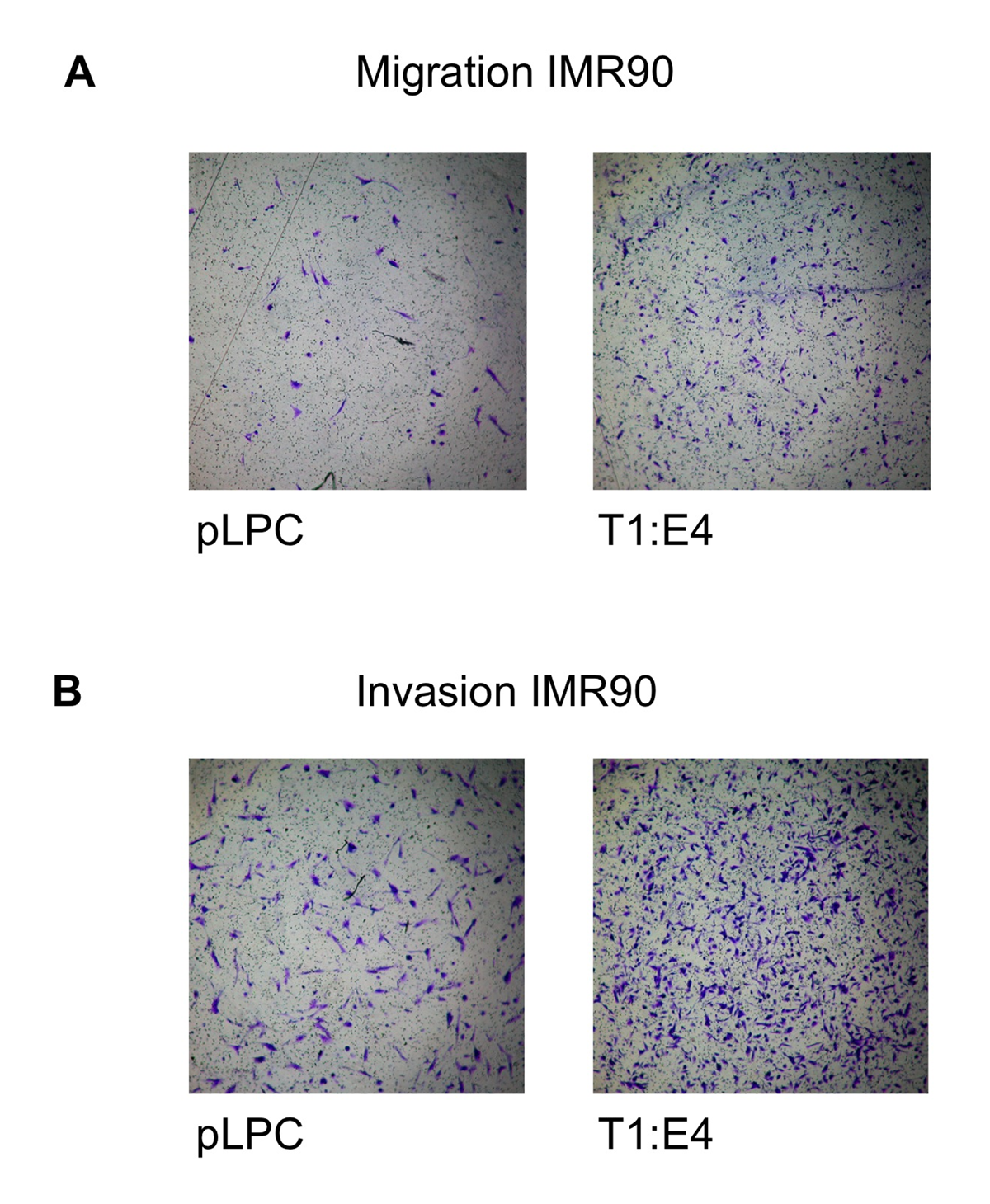

Supplement: Figure S2 — Effect of ERG 1b/T1:E4 ERG fusion variant on migration and invasion of IMR90 cells. Following drug selection, ERG 1b/T1:E4 -overexpressing clones or empty-vector control (pLPC) were assayed for their migration and invasion potential using a transwell migration (A) or matrigel invasion assay (B), as detailed in Material and Methods. (TIF) [file pone.0049721.s002.tif]

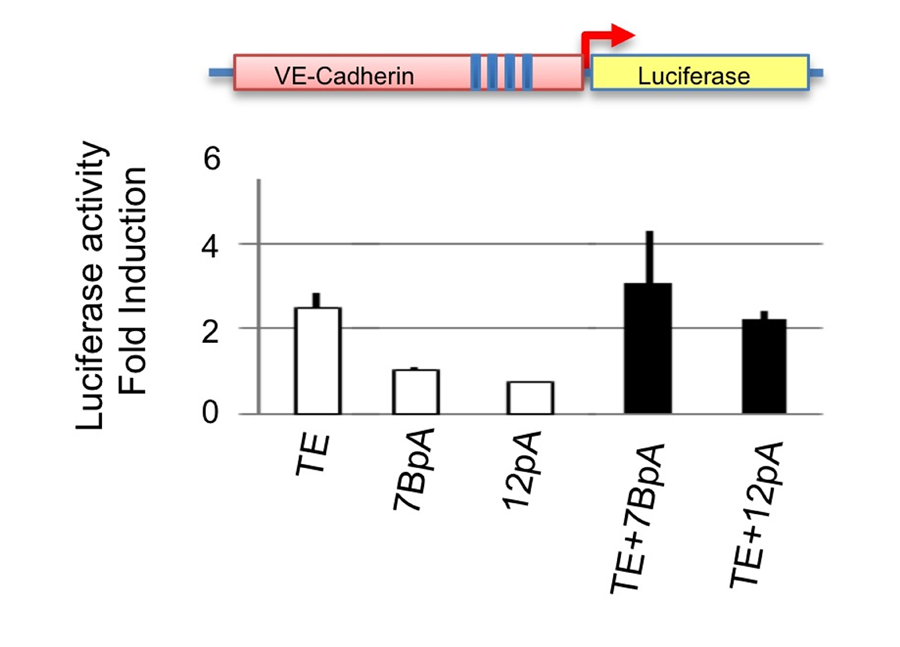

Supplement: Figure S3 — Activity of truncated fusion isoforms lacking Ets and TAD domains. Transient transcriptional activation of ERG-dependent VE-Cadherin promoter. The luciferase reporter was transiently co-expressed in HeLa cells with full-length T1:E4 or/and truncated TE:7bpA and TE:12pA variants (plus a Renilla luciferase vector to normalize for variation in transfection efficiency). Dual-luciferase assay was performed and activity is represented as fold-activity over that of co-transfected empty vector. Averages of at least 3 independent experiments, with standard deviations, are represented. The truncated isoforms alone cannot induce expression of luciferase, and when co-expressed together with the full-length protein they fail to inhibit its activity, ruling out for them a dominant-negative role. Further experiments would be required to reach more definitive conclusions. (TIF) [file pone.0049721.s003.tif]

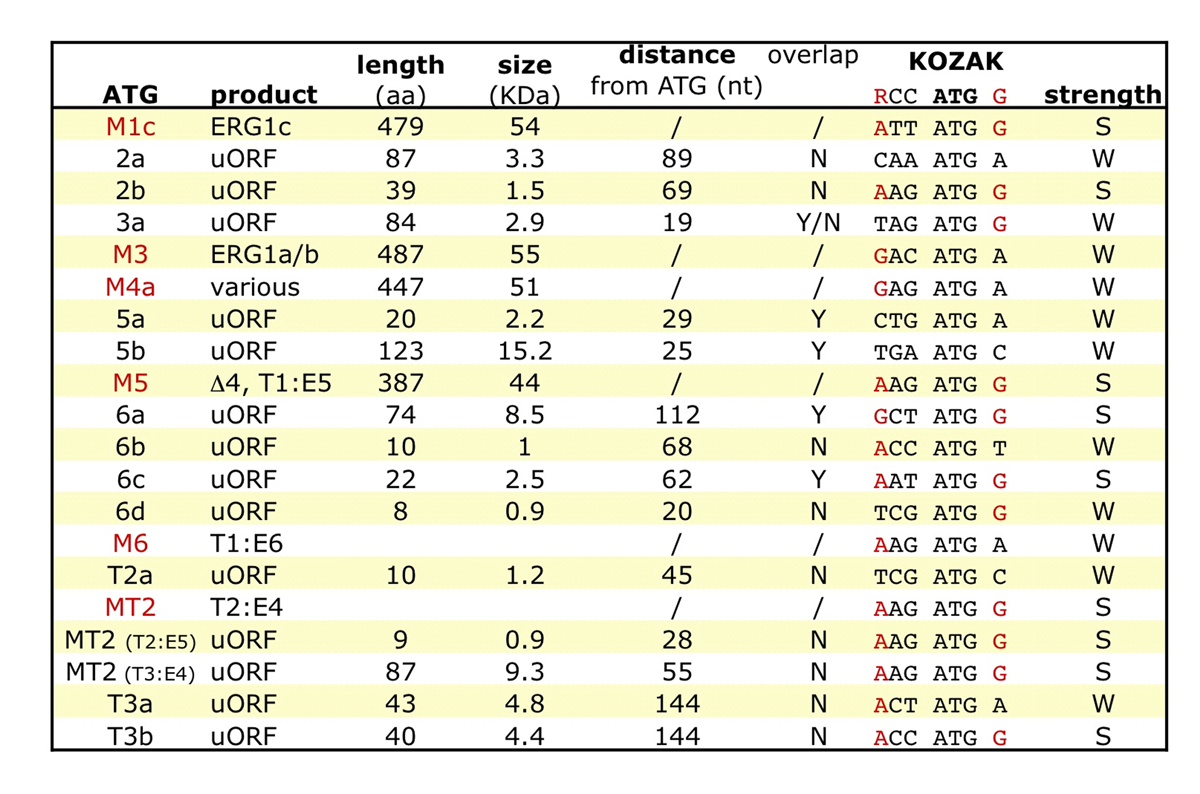

Supplement: Table S1 — Context and characteristics of uORF and in-frame ATG in ERG variants. ATGs from the 5′ region of various ERG variants are listed. ATG in frame with ERG are indicated in red and preceded by a ‘M’ ( = Met), ATG resulting in uORF are indicated in black, with the number indicating the exon that harbors them. The predicted length of the ORF/uORF (in amino acids and KDa) is reported, along with the distance (in nucleotides) from the translated ATG, whether the uORF overlaps with the translated ORF, and the ATG context. The context is considered ‘strong’, if both the determinant positions at −3 (G/A) and +4 (G) are conserved, and ‘weak’ if not. (TIF) [file pone.0049721.s004.tif]
